# Treatment pattern and clinical outcomes in multiple myeloma patients in Japan using the Medical Data Vision claims database

**DOI:** 10.1371/journal.pone.0283931

**Published:** 2023-04-06

**Authors:** Hiroshi Handa, Tadao Ishida, Shuji Ozaki, Asuka Mori, Kenichi Kato, Shinsuke Iida

**Affiliations:** 1 Department of Hematology, Gunma University Hospital, Gunma, Japan; 2 Department of Hematology, Japanese Red Cross Medical Center, Tokyo, Japan; 3 Department of Hematology, Tokushima Prefectural Central Hospital, Tokushima, Japan; 4 Integrated Market Access, Janssen Pharmaceutical K.K., Tokyo, Japan; 5 Medical Affairs, Janssen Pharmaceutical K.K., Tokyo, Japan; 6 Department of Hematology and Oncology, Nagoya City University Graduate School of Medical Sciences, Nagoya, Japan; Meiji Pharmaceutical University, JAPAN

## Abstract

Multiple myeloma therapy has made remarkable progress with the advent of new drugs. We explored the treatment pattern and outcomes in Japanese patients with multiple myeloma using the Medical Data Vision database. Patients were categorized as per the initial diagnosis period (2003–2015 and 2016–2020), considering the adoption of these new agents and then based on stem cell transplantation. Overall, 6438 patient data were extracted as eligible for data analysis, and the median age at the index diagnosis date was 72.0 years. Bortezomib/dexamethasone was the most common regimen for induction therapy in patients requiring stem cell transplantation from 2003–2015, and the use of bortezomib/lenalidomide/dexamethasone increased from 2016–2020. Lenalidomide/dexamethasone was the most commonly used post-transplant therapy. In the non-stem cell transplantation group, bortezomib/dexamethasone was mainly used for both periods, while lenalidomide/dexamethasone was primarily used from 2016–2020. There was a trend toward shorter first-line treatment duration and a shift to additional treatment patterns with new drugs at the following lines. The time to inpatient death period suggested an improvement between the two periods. Thus, this study revealed that recent diversification of treatment options is preferred and contributes to improved outcomes in the clinical practice of multiple myeloma in Japan.

## Introduction

Multiple myeloma (MM) is a hematologic malignancy characterized by the hyperproliferation of bone marrow plasma cells. It results in bone disease and includes lytic lesions and osteoporosis due to the suppression of osteoblast differentiation and increased osteoclast activity [1, 2]. The incidence of MM in Asia is reported as lower than that observed in global. However, recent reports have shown an increase in the morbidity rate in Asia [3]. In Japan, during 2016–2020, the age-adjusted incidence of MM increased from 5.9 to 6.1 per 100000 population. The incidence of MM increased with age, and the average age at diagnosis was 67 years [4, 5]. Although MM is an incurable disease, prolonged survival can be achieved with appropriate treatment, including supportive care, chemotherapy, and immunotherapy [2, 6].

For patients with newly diagnosed MM, treatment patterns are generally determined depending on patient characteristics (e.g., age, comorbidities, and physical condition) and the eligibility to undergo autologous stem cell transplantation (SCT) post induction therapy [2, 6]. The Japanese Society of Hematology recommends SCT for patients aged <65 years who have maintained major organ function. Patients aged ≥65 years, suffering from organ dysfunction, or any other risk factors associated with the immune system, are considered transplant-ineligible [7, 8]. In Japan, over the past several decades, therapeutic agents including bortezomib (approved in 2006), lenalidomide (approved in 2010), pomalidomide (approved in 2014), and thalidomide (approved in 2008) have demonstrated significant improvement in progression-free survival (PFS) and overall survival (OS) in patients with MM [9–12]. Additionally, several novel therapeutic agents have been introduced in Japan, such as panobinostat (approved in 2015), elotuzumab (approved in 2016), carfilzomib (approved in 2016), ixazomib (approved in 2016), daratumumab (approved in 2017), and isatuximab (approved in 2020) for the treatment of transplant-ineligible newly diagnosed patients and relapsed/refractory patients [2].

In the 2013 Japan Practical Guidelines for Hematological Malignancies, bortezomib/dexamethasone (VD) and bortezomib/cyclophosphamide/dexamethasone (VCD) were recommended as induction therapies for transplant-eligible patients [13]. Bortezomib/lenalidomide/dexamethasone (VRD) was newly added to the recommendation in the 2018 version [7], while in the latest 2020 version [14], VRD, VCD and VD were recommended. For transplant-ineligible patients, bortezomib/melphalan/prednisolone (VMP), melphalan/prednisolone (MP), and Vd were recommended in 2013 [13], which was updated to only recommend VMP and lenalidomide/dexamethasone (Rd; 18 cycles) in the 2018 version [7]. In the 2020 version, results of the two major studies, ALCYONE and MAIA study data, were considered, and daratumumab/bortezomib/melphalan/prednisolone (D-VMP) and daratumumab/lenalidomide/dexamethasone (D-Rd) were recommended [14].

A retrospective study conducted between 2008 and 2016 reported bortezomib with/without dexamethasone and MP regimens as the most preferred for the treatment of relapsed/refractory patients with MM in Japan [15]. However, studies on MM treatments covering the first line setting in Japan are sparse [2]. Considering a recent increment in the novel treatment options for MM in Japan, a comprehensive report on current treatment patterns and outcomes is required. Such information is vital for continuous improvement in the management of MM to achieve optimal patient care and to understand the disease burden and healthcare services available for patients with MM in Japan in a real-world setting [2]. The present study examined treatment patterns of each MM drug and the treatment outcomes for the age at diagnosis and transplantation using a health insurance claims database in Japan.

## Methods

### Data source

This was a retrospective, longitudinal, observational cohort study in patients with MM. Retrospective claims data from the Medical Data Vision (MDV) database were analyzed from 01 January 2003 to 31 May 2021. The MDV database, comprising standardized healthcare insurance claims data provided by hospitals in Japan, has been utilizing the Japanese Diagnosis and Procedure Combination (DPC) fixed-payment reimbursement system for over 36 million individuals since 2003; it covers about 30000 patients with MM. Under the DPC system, treatments such as basic hospital stays, tests, and diagnostic imaging are reimbursed with a comprehensive payment set for different diagnosis groups. Treatments such as surgery and anesthesia are reimbursed based on a fee-for-service system.

### Study design and patient population

Adult patients aged ≥20 years with a diagnosis of MM between 2003 and 2020, as per data available in the MDV database, were considered for this study. MM diagnosis was defined as the presence of at least one record with a confirmed MM diagnosis code (International Classification of Diseases [ICD]-10 code C90.0 in combination with the Japanese disease codes (2030003, 8839397, 8840039, 8842090, 8847152, 8847175, 8847250, and 8847258). The index diagnosis date was defined as the date on which the patient had the first record of a confirmed MM diagnosis. The baseline period was 12 months before the index diagnosis date. The follow-up period was ≥60 days from the index diagnosis date; however, patients who died within these 60 days were followed for <60 days.

Patients must also have continuous care in the baseline and follow-up periods. Those with an SCT record were included in the analysis even if they had a baseline period of <12 months. Patients with evidence of MM, other primary cancer(s), or metastatic disease in the baseline period were excluded from the study.

**Fig 1:**
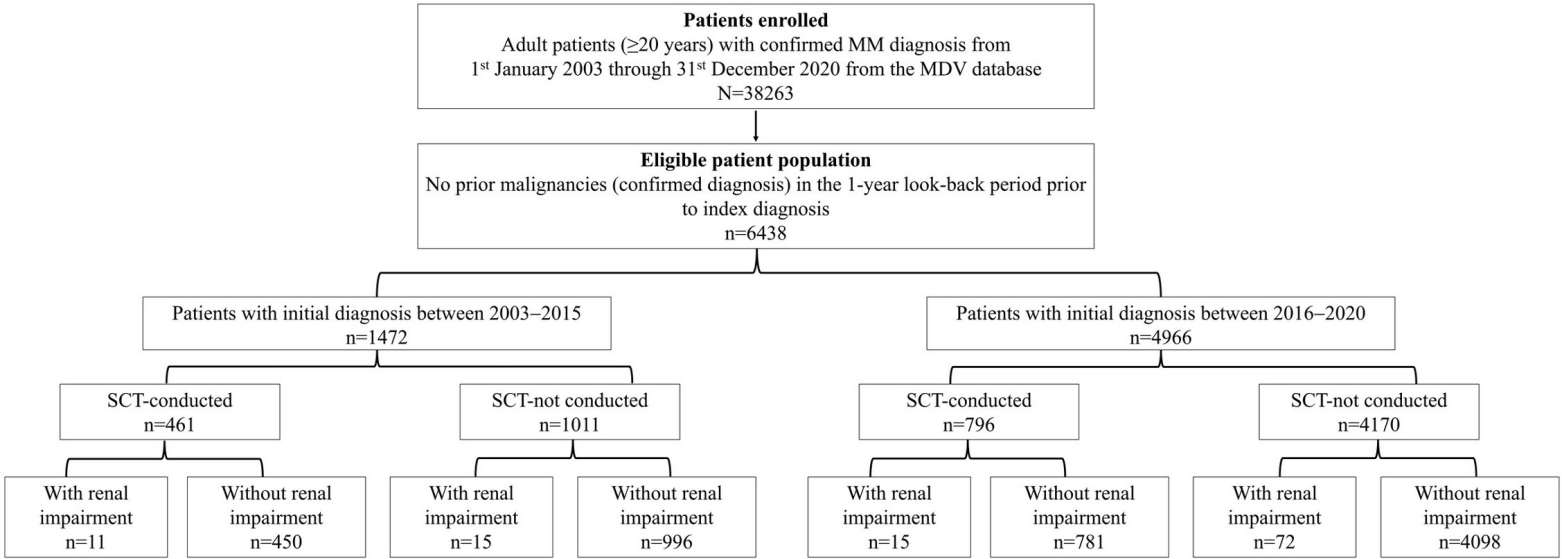
Study population flowchart. MDV, Medical Data Vision; SCT, stem cell transplantation. **Note:** ICD10 codes I12.0, I13.1, N03.2 -N03.7, N05.2 -N05.7, N18.x, N19.x, N25.0, Z49.0-Z49.2, Z94.0, and Z99.2 were selected to identify renal impairment.

Patients were categorized into two groups based on the time of their initial diagnosis: 1) between 2003 and 2015 and 2) between 2016 and 2020. These categorizations were also executed considering approval of several new therapeutic agents for treatment multiple myeloma in Japan in 2015. Patients were subsequently divided into subgroups based on SCT after the index date (conducted and not conducted) and the presence of renal impairment during the baseline period (Fig 1). This analysis didn’t have the review from the Institutional Review Board since it includes the anonymized claims data from secondary database.

### Study outcomes

The main objective of the study was to evaluate treatment patterns in patients with MM receiving routine clinical care in Japan using the MDV database. Data were reported for different sub-groups as described above. Baseline demographics and clinical characteristics (age, sex, year of MM diagnosis, comorbidities, etc.) were reported. The proportion of the treatment regimens for each line (1L to 6L) was assessed and reported for different subgroups.

Duration of therapy was defined as the time from the initiation to the last day of the line of treatment. A regimen was established with at least one date of administration of the first drug following its administration in the follow-up; the interval between the dates fell within the range of the grace periods set up for each drug, considering the days of prescription. Additional drugs for the regimen were identified if another drug was administered within 28 days of the drug’s first administration. The date of administration of the target drug regimen was prescribed at least once after the date of the first administration, and the interval was within the range of the grace period for the drug. Switching to a new regimen was identified if a drug that was not the target drug in the previous regimen was newly administered at least once after the first administration date, and the interval between the two dates fell within the grace period of the drug.

The line of therapy (LoT) was considered as ended in one of the following conditions: (1) initiation of a new regimen; (2) 14 days or longer for any drug considering the grace period; (3) death or the end of the data. Attrition rates, defined as the percentage of patients who dropped out of the previous line, were also assessed, and reported for each line of treatment. Time to inpatient death was assessed from the index date through the date of the death of any cause. Those patients whose data ended without the death record were treated as ’censored’ at the end of the follow-up. Considering death information was only available from hospitalized patients, time to inpatient death was calculated only for such patients. For other patients, the duration to the last observation date was calculated and was handled as the right-censored value.

### Statistical analyses

For baseline demographics, clinical characteristics, and treatment pattern data, descriptive summaries were provided. For survival data, the summary statistics (minimum, 25^th^ percentile, median, 75^th^ percentile, maximum) for time to inpatient death were presented, along with the Kaplan-Meier plot.

## Results

### Baseline demographic and clinical characteristics

A total of 38370 patients with MM were registered in the MDV database, and 6438 (16.78%) were considered eligible; 53.4% (n = 3441) of these were men. The median (minimum-maximum) age at the index diagnosis date was 72.0 (20.0–100.0) years; 43.36% of patients were ≥75 years of age at the index diagnosis date (Table 1). Of the eligible patients, 1472 (22.9%) had an index diagnosis year between 2003 and 2015, and 4966 (77.1%) had between 2016 and 2020 (Table 1 and Fig 1). A total of 1257 patients were included in the SCT-conducted group, and 5181 were in the SCT not-conducted group (Table 1 and Fig 1). In the SCT-conducted group, 632 (50.3%) patients were aged <60 years at the index date, 331 (26.3%) were aged 60–64 years, 261 (20.8%) were aged 65–69 years, and 33 (2.6%) were aged ≥70 years (Table 1). In the SCT not-conducted group, 3632 (70.1%) patients were aged ≥70 years at the index date. Overall, the mean (standard deviation [SD]) Charlson Comorbidity Index (CCI) score was 0.7 (1.35), with 0.1 (0.52) in the SCT-conducted group without renal impairment and 0.8 (1.45) in the SCT not-conducted group without renal impairment (Table 1).

**Table 1:** Baseline demographic and clinical characteristics.

Parameters	Eligible population	SCT-conducted group		SCT not-conducted group	
		With renal impairment	Without renal impairment	With renal impairment	Without renal impairment
**N**	6438	26	1231	87	5094
**Female, n (%)**	2997 (46.6)	13 (50.0)	541 (43.9)	51 (58.6)	2392 (47.0)
**Male, n (%)**	3441 (53.4)	13 (50.0)	690 (56.1)	36 (41.4)	2702 (53.0)
**Index Year, n (%)**					
**2003–2015**	1472 (22.9)	11 (42.3)	450 (36.6)	15 (17.2)	996 (19.6)
**2016–2020**	4966 (77.1)	15 (57.7)	781 (63.4)	72 (82.8)	4098 (80.4)
**Age, Mean (SD) (Median [min-max])**	70.5 (12.92) (72.0 [20.0−100.0])	59.1 (7.41) (60.5 [43.0−73.0])	57.9 (8.18) (59.0 [21.0−75.0])	76.5 (10.19) (79.0 [38.0−96.0])	73.4 (12.01) (75.0 [20.0−100.0])
**Age groups, years**					
**18–59**	1238 (19.2)	12 (46.2)	620 (50.4)	5 (5.7)	601 (11.8)
**60–64**	625 (9.7)	9 (34.6)	322 (26.2)	4 (4.6)	290 (5.7)
**65–69**	910 (14.1)	4 (15.4)	257 (20.9)	13 (14.9)	636 (12.5)
**70–74**	873 (13.6)	1 (3.8)	30 (2.4)	9 (10.3)	833 (16.4)
**75–79**	1063 (16.5)	0	2 (0.2)	17 (19.5)	1044 (20.5)
**80–84**	911 (14.2)	0	0	21 (24.1)	890 (17.5)
**85–89**	598 (9.3)	0	0	15 (17.2)	583 (11.4)
**90–94**	188 (2.9)	0	0	1 (1.1)	187 (3.7)
**95–99**	30 (0.5)	0	0	2 (2.3)	28 (0.5)
**100-**	2 (0.0)	0	0	0	2 (0.0)
**CCI Score, Mean (SD) [Median (min-max)]**	0.7 (1.35) (0.0 [0.0−9.0])	0.3 (0.78) (0.0 [0.0−3.0)]	0.1 (0.52) (0.0 [0.0−7.0])	0.6 (1.01) (0.0 [0.0−4.0])	0.8 (1.45) (0.0 [0.0−9.0])
**0**	4471 (69.4)	23 (88.5)	1148 (93.3)	57 (65.5)	3243 (63.7)
**1**	804 (12.5)	0	39 (3.2)	14 (16.1)	751 (14.7)
**2**	511 (7.9)	2 (7.7)	28 (2.3)	10 (11.5)	471 (9.2)
**3**	293 (4.6)	1 (3.8)	13 (1.1)	4 (4.6)	275 (5.4)
**4+**	359 (5.6)	0	3 (0.2)	2 (2.3)	354 (6.9)

CCI, Charlson Comorbidity Index; SCT, stem cell transplantation; SD, standard deviation

### Regimen selection and duration of treatment

**Fig 2:**
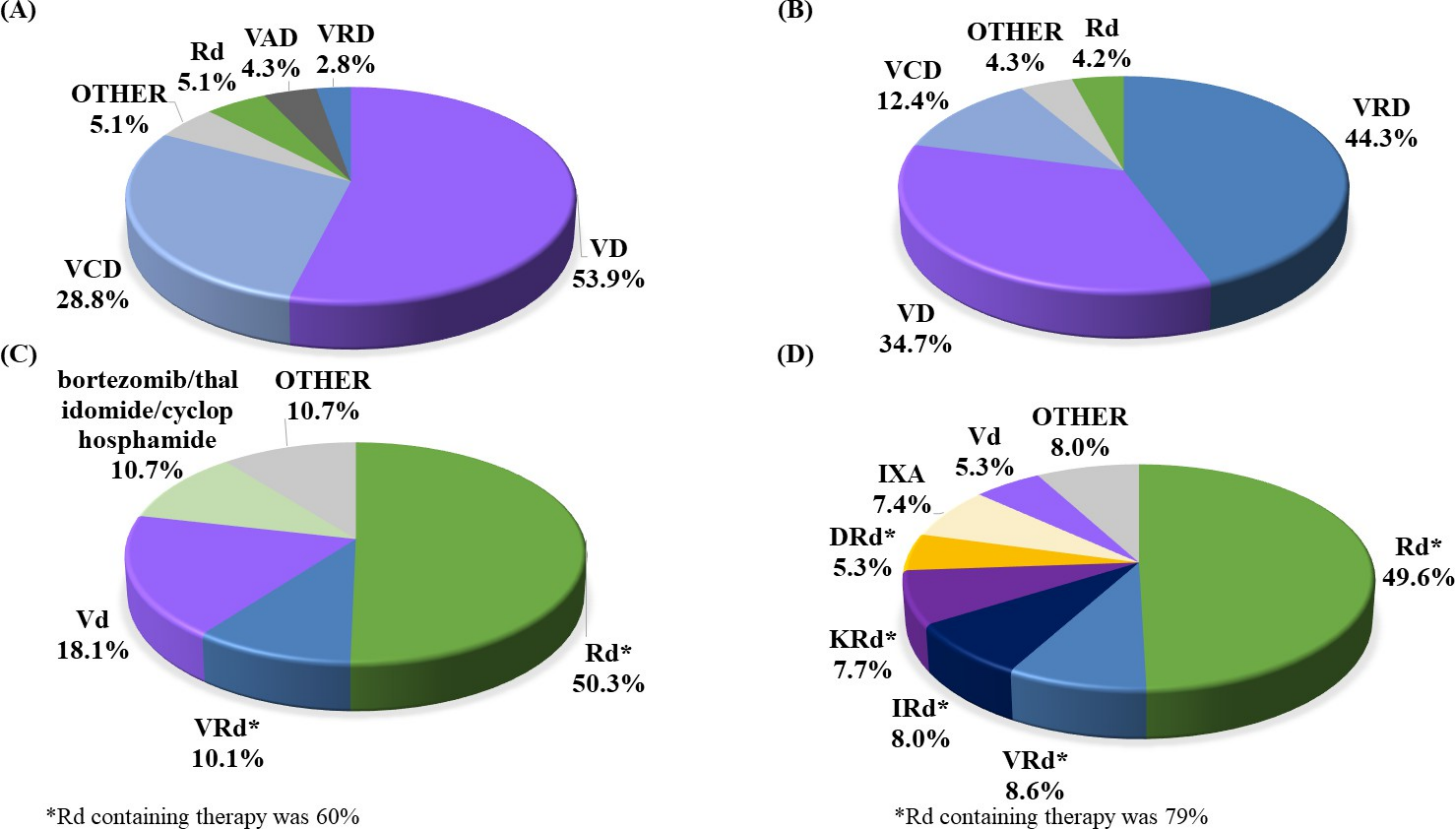
Regimen selection for induction therapy before SCT and post-SCT therapy in the SCT-conducted group. (**A**) 2003 to 2015 induction therapy before SCT, (**B**) 2016 to 2020 induction therapy before SCT, (**C**) 2003 to 2015 post-SCT therapy, (**D**) 2016 to 2020 post-SCT therapy. DRd, daratumumab/lenalidomide/dexamethasone; IRd, ixazomib/lenalidomide/dexamethasone; IXA, ixazomib; KRd, carfilzomib/lenalidomide/dexamethasone; Rd, lenalidomide/dexamethasone; SCT, stem cell transplantation; VAD/VAd, vincristine/doxorubicin/dexamethasone; VCD/VCd, bortezomib/cyclophosphamide/dexamethasone; VD/Vd, bortezomib/dexamethasone; VRD/VRd, bortezomib/lenalidomide/dexamethasone. **Note:** Post-SCT therapy is the initiation date of the regimen between the date of SCT and post 4 months from SCT.

In the SCT-conducted group, before SCT, VD (53.9% of patients) was the commonly selected induction regimen in the 2003–2015 cohort, and VRD (44.3% of patients) in the 2016–2020 cohort, (Fig 2A and 2B). The duration of VD treatment tended to be short, from a median of 82 days (2003–2015) to a median of 53 days (2016–2020), and in recent cohorts, some patients were changed to VCD, VRD, and Rd treatment. VRD induction increased in recent cohorts, but it was confirmed that bortezomib and lenalidomide were discontinued in about 3% of patients, and many cases continued. The median duration of treatment was 99 days (S1 Fig in S1 File).

For post-SCT therapy, Rd was the most common regimen in the patients treated by post-SCT therapy, utilized by 50.3% of patients (2003–2015) and 49.5% of patients (2016–2020) (Fig 2C and 2D).

**Fig 3:**
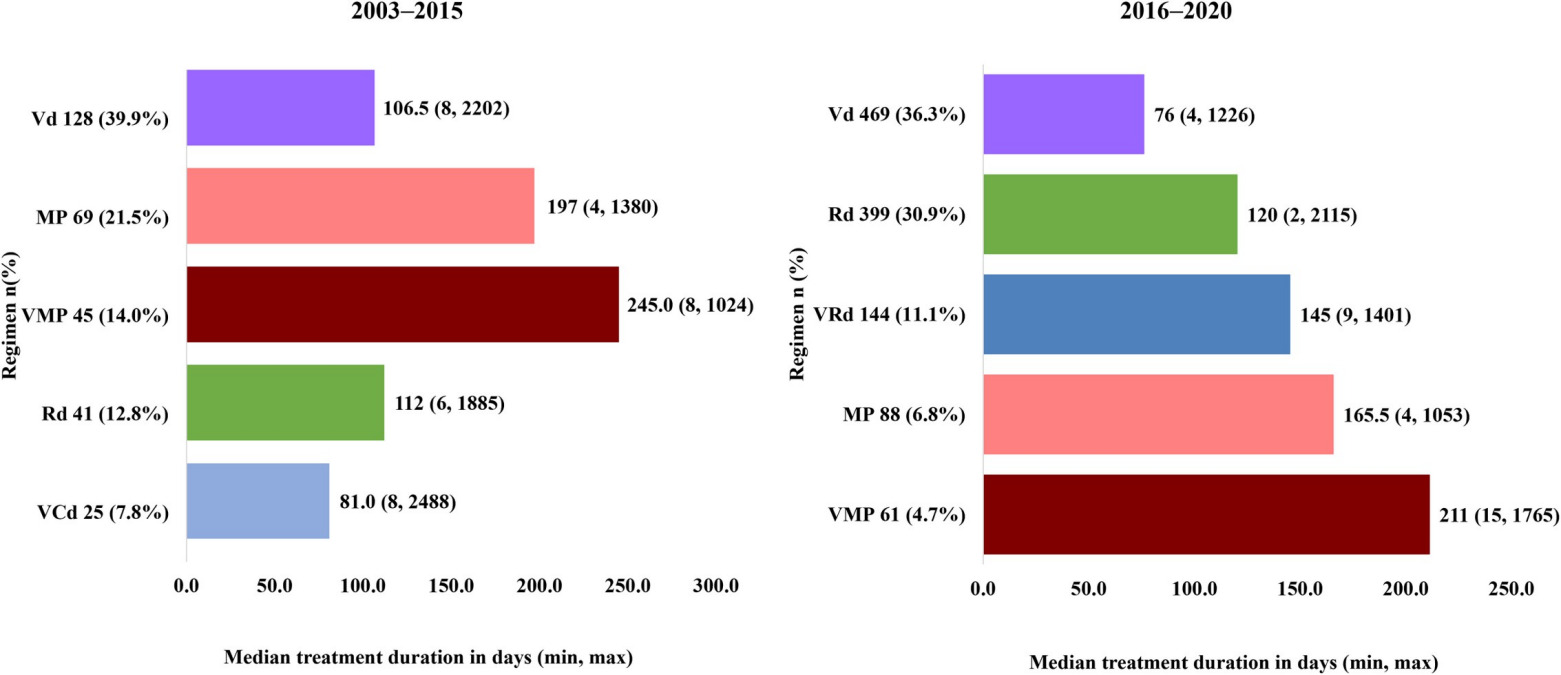
First-line treatment duration by selected regimen in SCT not-conducted group (All population). MP, melphalan/prednisolone; Rd, lenalidomide/dexamethasone; VCd, bortezomib/cyclophosphamide/dexamethasone; Vd, bortezomib/dexamethasone; VMP, bortezomib/melphalan/predonisolone; VRd, bortezomib/lenalidomide/dexamethasone. **Note:** Top 5 regimens in the proportion of use were selected.

In the SCT not-conducted group, Vd was the most selected regimen in 39.9% of patients from 2003–2015 and 36.3% from 2016–2020. Additionally, MP in the 2003–2015 cohort (21.5%) and Rd in the 2016–2020 cohort (30.9%) were the standard regimens used in the SCT not-conducted group (Fig 3).

**Fig 4:**
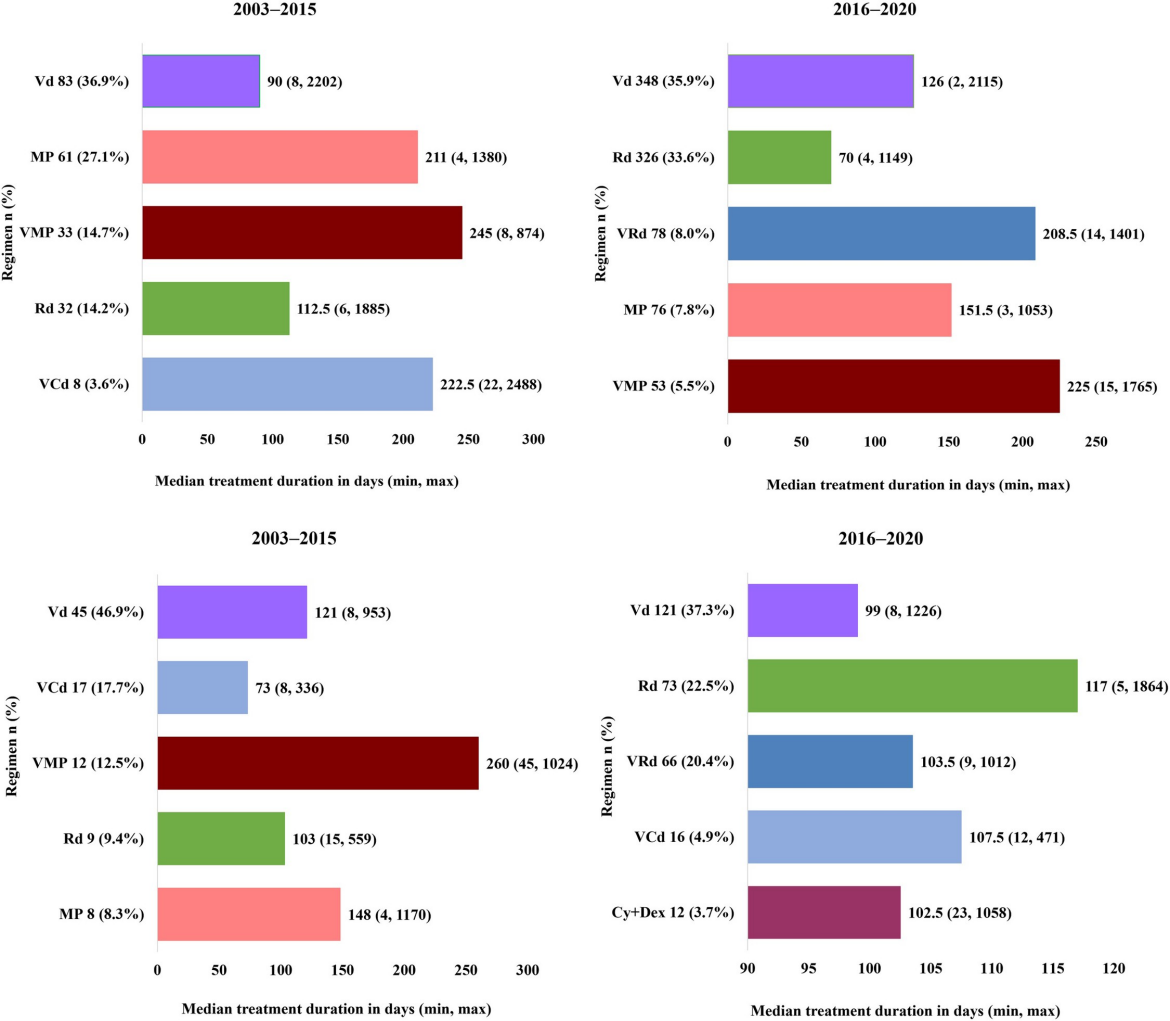
**A**: First-line treatment duration by selected regimen in SCT not-conducted group (aged >70 years). **B**: First-line treatment duration by selected regimen in SCT not-conducted group (aged ≤70 years). Cy+Dex, cyclophosphamide/dexamethasone; MP, melphalan/prednisolone; Rd, lenalidomide/dexamethasone; VCd, bortezomib/cyclophosphamide/dexamethasone; Vd, bortezomib/dexamethasone; VMP, bortezomib/melphalan/predonisolone; VRd, bortezomib/lenalidomide/dexamethasone. **Note:** Top 5 regimens in the proportion of use were selected.

Regarding treatment duration in the overall SCT not-conducted group, the treatment duration was shorter in the 2016–2020 cohort than in the 2003–2015 cohort (Fig 3 and S2A Fig in S1 File). Treatment duration by age (>70 years and ≤70 years, Fig 4) analysis in the SCT not-conducted group demonstrated a similar trend in both the age cohorts and in >80 years (S2B Fig in S1 File).

In the SCT not-conducted group, Vd was the most commonly selected treatment regimen in 38.5% (2003–2015) and 57.1% (2016–2020) of patients with renal impairment (S3A Fig in S1 File) and 40.0% (2003–2015) and 34.2% (2016–2020) of patients without renal impairment (S3B Fig in S1 File). Regimen selection data for the second line (2L) and later treatments were available for the 2003–2015 and the 2016–2020 cohorts (S4A and S4B Fig in S1 File). Attrition rates by the line of therapy (for the 2003–2015 and the 2016–2020 cohorts) are available in Table 2. The percentage of death was lower with early lines of treatment (1L to 4L) in the 2016–2020 cohort than in the 2003–2015 cohort. However, the attrition rate increased with each line of treatment; the treatment duration became shorter in the 2016–2020 cohort than in the 2003–2015 cohort (Table 2).

**Table 2:** Attrition rates by the line of therapy (2003–2015, and 2016–2020).

2003–2015							
LoT	Frequency	% Attrition	Death, n (%)	Lost to follow-up, n (%)	No subsequent treatment in follow-up, n (%)	Subsequent Treatment, n (%)	Mean treatment duration Month [SD] (median)
**1**	740	-	39 (5.3)	36 (4.9)	155 (20.9)	510 (68.9)	10.0 [12.08] (6.0)
**2**	510	31.1	20 (3.9)	57 (11.2)	71 (13.9)	362 (71.0)	11.3 [13.17] (5.8)
**3**	377	26.1	26 (6.9)	52 (13.8)	45 (11.9)	254 (67.4)	8.8 [10.21] (5.1)
**4**	254	32.6	22 (8.7)	33 (13.0)	24 (9.4)	175 (68.9)	6.2 [7.08] (3.9)
**5**	175	31.1	14 (8.0)	24 (13.7)	18 (10.3)	119 (68.0)	6.8 [8.95] (3.2)
**6**	119	32.0	16 (13.4)	19 (16.0)	12 (10.1)	72 (60.5)	5.1 [6.57] (3.1)
**2016–2020**							
**1**	2033	-	97 (4.8)	399 (19.6)	498 (24.5)	1039 (51.1)	8.6 [10.10] (4.8)
**2**	1039	48.9	44 (4.2)	276 (26.6)	132 (12.7)	587 (56.5)	7.3 [8.41] (3.9)
**3**	643	38.1	43 (6.7)	171 (26.6)	91 (14.2)	338 (52.6)	6.7 [7.83] (3.7)
**4**	338	47.4	19 (5.6)	123 (36.4)	33 (9.8)	163 (48.2)	6.5 [8.34] (3.4)
**5**	163	51.8	16 (9.8)	42 (25.8)	16 (9.8)	89 (54.6)	4.4 [5.31] (2.8)
**6**	89	45.4	13 (14.6)	28 (31.5)	8 (9.0)	40 (44.9)	4.0 [4.40] (2.3)

LoT, line of treatment; SD, standard deviation

### Time to inpatient death

**Fig 5:**
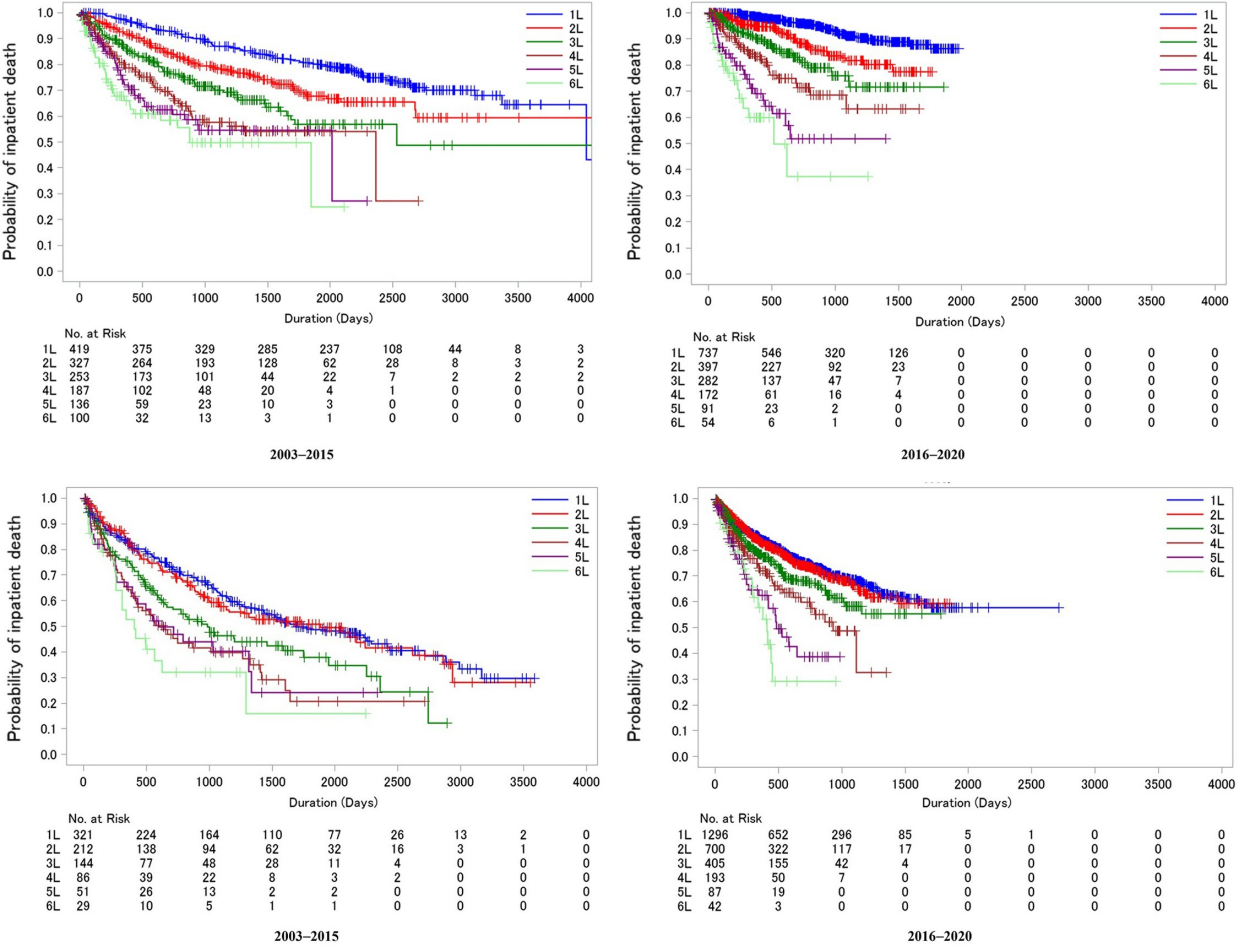
**A**. Kaplan-Meier curve for time to inpatient death by the lines of treatment for SCT-conducted group in 2003–2015 and 2016–2020 cohorts. **B.** Kaplan-Meier curve for time to inpatient death by the lines of treatment for SCT not-conducted group in 2003–2015 and 2016–2020 cohorts. SCT, stem cell transplantation.

Kaplan-Meier time to inpatient death curves per different lines of treatment in the SCT-conducted and not-conducted groups are available in Fig 5A and 5B. For the SCT-conducted group, the median time to inpatient death from the treatment initiation of 1L to 6L was 4043, not estimated (NE), 2531, 2363, 2013, and 880 days, respectively, in the 2003–2015 cohort. The median time to inpatient death from each treatment line’s initiation was NE for 1L to 5L and shorter in 6L as 519 days for 2016–2020 cohort than the 2003–2015 cohort. For the SCT not-conducted group, the median time to inpatient death from the treatment initiation of 1L to 6L was 1684, 1882, 986, 643, 599, and 406 days, respectively, in the 2013–2015 cohort. The median time to inpatient death from the treatment initiation of 1L to 3L was NE; for 4L, 5L, and 6L, it was 932, 486, and 412 days in the 2016–2020 cohort.

## Discussion

The current retrospective insurance claims database study described treatment patterns and outcomes in patients with MM in a real-world setting in Japan. Remarkable treatment progress has been observed with novel agents, and SCT remains the backbone for transplant-eligible patients [16]. In the current study, the proportion of male patients was moderately high, in line with the epidemiological data in Japan [5]. Most of the patients included in the SCT-conducted group were aged <65 years, which is consistent with the recommendation of the Japanese Society of Hematology to carry out SCT in patients with MM aged <65 years and eligible for transplantation [7, 8]. Both SCT-eligible and SCT-ineligible patients with MM experience relapse and hence require subsequent lines of therapy [6]. Consistent with results obtained from previously conducted MM studies in Japan, bortezomib-based regimens, melphalan-prednisone-like regimens, and lenalidomide-based regimens were the most commonly prescribed subsequent LOTs [8, 15]. The trend towards a shorter duration of treatment over recent years, as reported in this study, was similar to local epidemiological data from Japan which reported the time to subsequent MM therapy as 20.2 months for the patients who initiated the treatment until 2010, 10.0 months for those who initiated treatment between 2011 to 2015, and 3.8 months for ones who initiated treatment between 2016 to 2020; this data can be regarded as reasonably relevant [17].

A study from the Intergroupe Francophone du Myélome reported Vd as the most frequently used induction therapy. The response rate was 67%, with 31% of patients achieving good partial response and 21% with complete response or near complete remission [6]. Similarly, in the current study, Vd and VRd were the most selected regimens in the SCT-conducted group as induction therapy, consistent with previous studies in Japan for MM [18]. In the 2003–2015 cohort, for most patients who initiated induction from Vd or VCd, the regimen was changed in approximately one-third of patients. In the 2016–2020 cohort, most VRd-induced patients proceeded to SCT without any additional or altered regimen. The induction therapy was changed to VRd in most patients who did not initiate the induction from VRd, and one-third of patients who initiated the induction therapy by Vd changed the treatment to VRd as the subsequent induction therapy.

Post-SCT therapy, Rd and Rd-containing regimens were reported to be the most common in the current study. The JSCT-MM12 and MM14 studies conducted by the Japan Study Group for Cell Therapy and Transplantation suggested lenalidomide as one of the effective options for maintenance therapy after SCT [19, 20]. Significantly prolonged progression-free survival was achieved with lenalidomide maintenance therapy post-SCT in a randomized, phase 3 clinical trial conducted at 77 study centers in Switzerland, Belgium, and France [21]. In the SCT not-conducted group, Vd was the most selected regimen, followed by MP and Rd in the current study. In the Japan Practical Guidelines for Hematological Malignancies, VMP and Rd (18 cycles) were recommended in 2018 [7], and D-VMP and D-Rd were added to the recommendation in 2020 [14]. The reason for the discrepancy from the clinical guideline recommendation is that the dose intensity from the VISTA trial regimen (VMP) was not considered fit for Japanese patients; therefore, VMP was not frequently used.

With an early line of treatment (1L to 4L) in our study, the percentage of patients who died decreased in the 2016–2020 cohort compared with the 2003–2015 cohort. The attrition rate increased with each line of treatment, and the treatment duration became shorter in the 2016–2020 cohort compared with the 2003–2015 cohort. This shorter treatment duration might reflect the gradual spread of the shift towards triple therapy in our study. The triple therapy included newly available drugs those were approved after 2015, particularly in relapsed/refractory MM. Recent clinical studies and meta-analyses reported that triple therapy including newly approved drugs (Carfilzomib, Ixazomib, Elotuzumab, and Daratumumab) are associated with superior efficacy versus dual therapy [22–31]. The shift to the triple therapy is carried out with the aim of obtaining higher efficacy. Although the duration of treatment before switching is shortened, improved outcomes are expected.

The availability of novel agents to treat MM in Japan increased during 2003–2020 and was prescribed to patients aged <65 years and ≥65 years, suggesting an improvement in the estimated OS [2, 6]. Ozaki et al. reported that using novel agents prolonged OS in both the SCT-conducted and SCT not-conducted groups [32]. Besides, findings from three MM registries in the US also suggested proportionate improvement in OS rates between 2010 and 2015 [33]. This current study suggested an improvement in time to inpatient death within a year of diagnosis in both the SCT-conducted and SCT not-conducted groups during 2016–2020 and was consistent with previous studies. In particular, 2L in the non-SCT group may be affected by adding new drugs or switching.

The study had some limitations. Findings may not represent the entire population in Japan as the available data are limited to the claims database. Death was captured only for hospitalized patients, and critical prognosis variables for outcomes such as baseline molecular, and cytogenetics were unavailable. Furthermore, it was challenging to identify comorbidities and complications as some diseases (e.g., mild increase in creatinine from renal impairment, bone lesions, etc.) were not recorded as administrative claims. As reported previously [34], the information on death from hospitalized patients in the JMDC database was compared with the registry data. The sensitivity of the anti-cancer drug prescription for patients to predict hospital death concerning actual death was as high as 90.7%. We expected similar sensitivity for the patient’s death information from the discharge summary in this study. Although inpatient death is a valuable clinical outcome, this analysis was focused to highlight the turning point due to the shortening of treatment duration with the widespread adoption of new drugs. Despite the above limitations, the study covered a large patient population, reflecting the most recent therapeutic agent approved in Japan for the treatment of MM, representative of the real-world clinical setting.

## Conclusion

The current study evaluated treatment patterns and clinical outcomes in patients with MM using the MDV claims database in Japan. The claims data provided real-world information on the presence/absence of transplantation, initiation time-to-treatment, regimen selection by age, and the duration of treatment. Further investigations are required to characterize treatment patterns and outcomes in individuals who underwent SCT. Although it was difficult to clarify the characteristics of treatment changes in the era of new drugs, our study suggested an improvement in survival time for both the SCT-conducted and the SCT not-conducted groups over time. This study showed the improvement of treatment outcomes along with the recent diversification of treatment options.

## Supporting information

S1 File Supplementary figures (S1 Fig to S4 Fig). (DOCX)
