# Supplementary material for: Treatment pattern and clinical outcomes in multiple myeloma patients in Japan using the Medical Data Vision claims database
Source: PLoS One. 2023 Apr 6;18(4):e0283931. doi: 10.1371/journal.pone.0283931 (PMC10079007; doi:10.1371/journal.pone.0283931)
Supplement: S1 File — (DOCX) [file pone.0283931.s001.docx]

**Supporting information**

**Figure S1:** Regimen selections for the induction and re-induction therapy before SCT in the SCT-conducted group


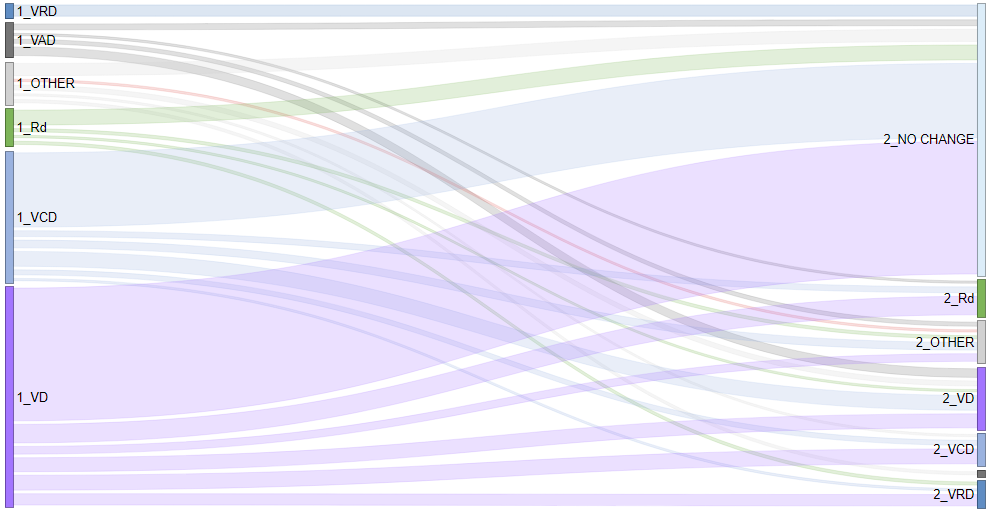


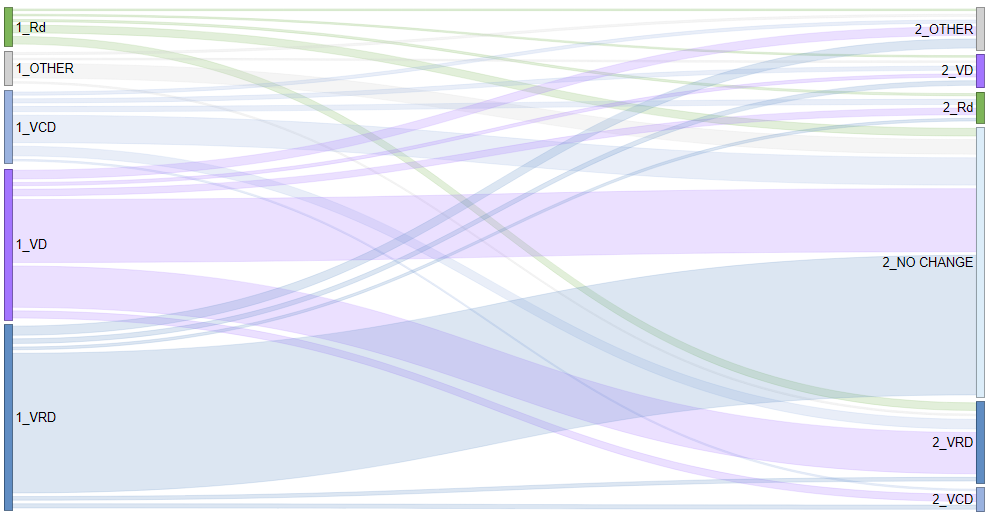


Cy+Dex, cyclophosphamide/dexamethasone; MP, melphalan/prednisolone; RD, lenalidomide/dexamethasone; VAD, vincristine/doxorubicin/dexamethasone; VCD, bortezomib/cyclophosphamide/dexamethasone; VD, bortezomib/dexamethasone; VMP, bortezomib/melphalan/prednisolone; VRD, bortezomib/lenalidomide/dexamethasone

**Figure S2A**: First-line treatment duration by selected regimen in SCT not-conducted group (aged >75 years)


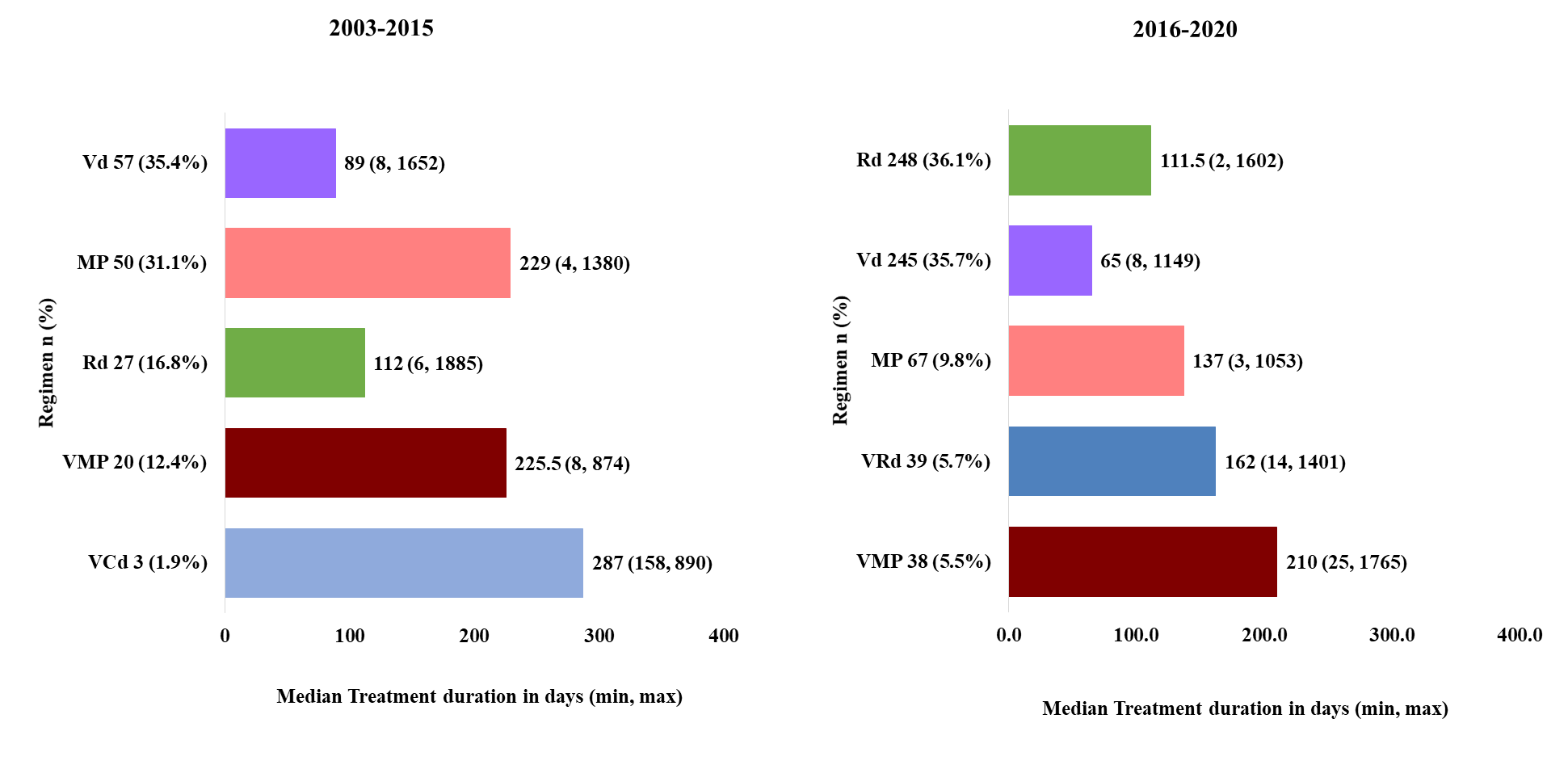


MP, melphalan/prednisolone; Rd, lenalidomide/dexamethasone; VCd, bortezomib/cyclophosphamide/dexamethasone; Vd, bortezomib/dexamethasone; VMP, bortezomib/melphalan/prednisolone; VRd, bortezomib/lenalidomide/dexamethasone

**Note:** Top 5 regimens in the proportion of use were selected.

**Figure S2B**: First-line treatment duration by selected regimen in SCT not-conducted group (aged >80 years)


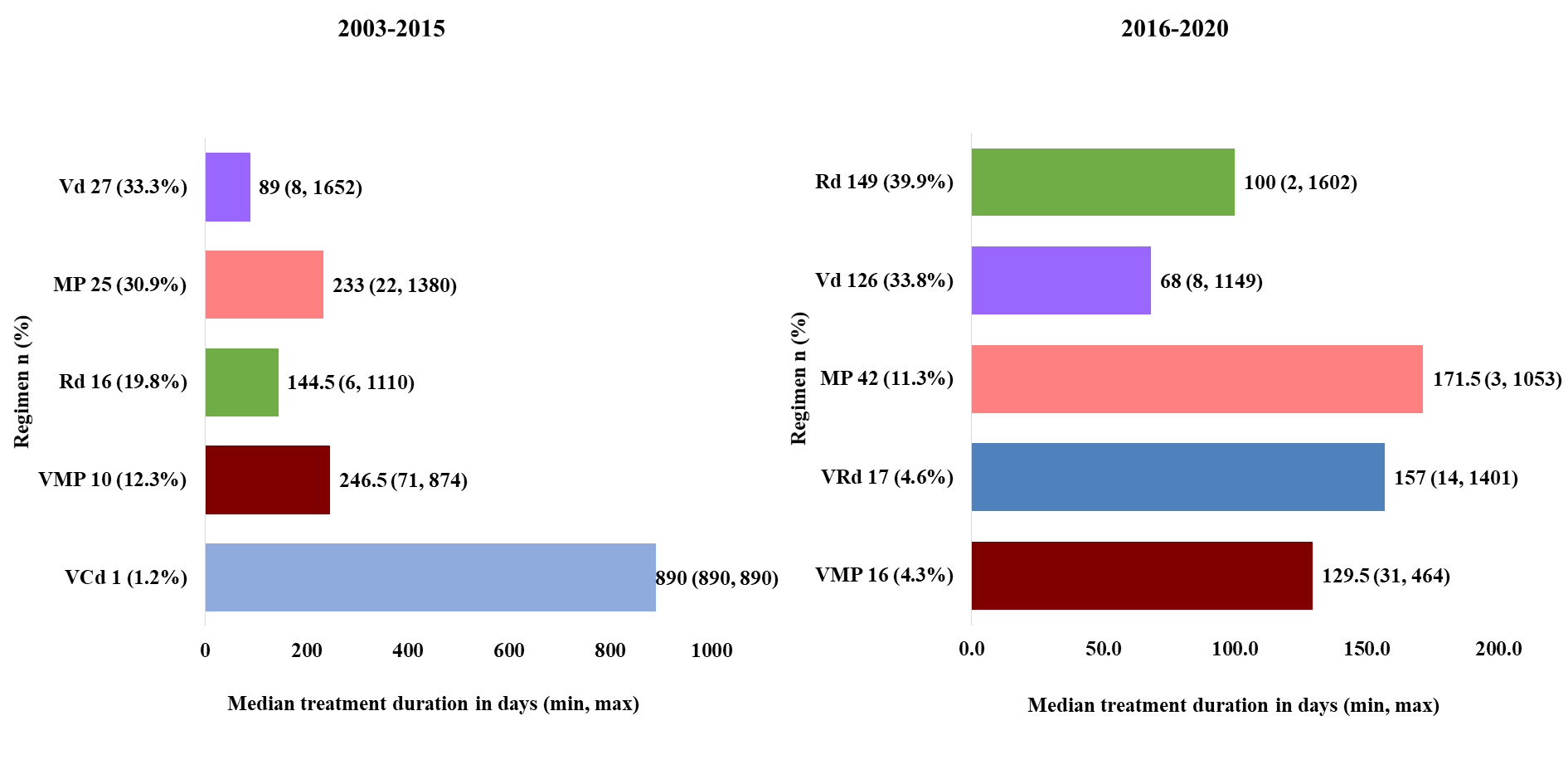
MP, melphalan/prednisolone; Rd, lenalidomide/dexamethasone; VCd, bortezomib/cyclophosphamide/dexamethasone; Vd, bortezomib/dexamethasone/ prednisolone; VMP, bortezomib/melphalan; VRd, bortezomib/lenalidomide/dexamethasone

**Note:** Top 5 regimens in the proportion of use were selected.

**Figure S3A:** First-line treatment duration by selected regimen in SCT not-conducted group (with renal impairment)


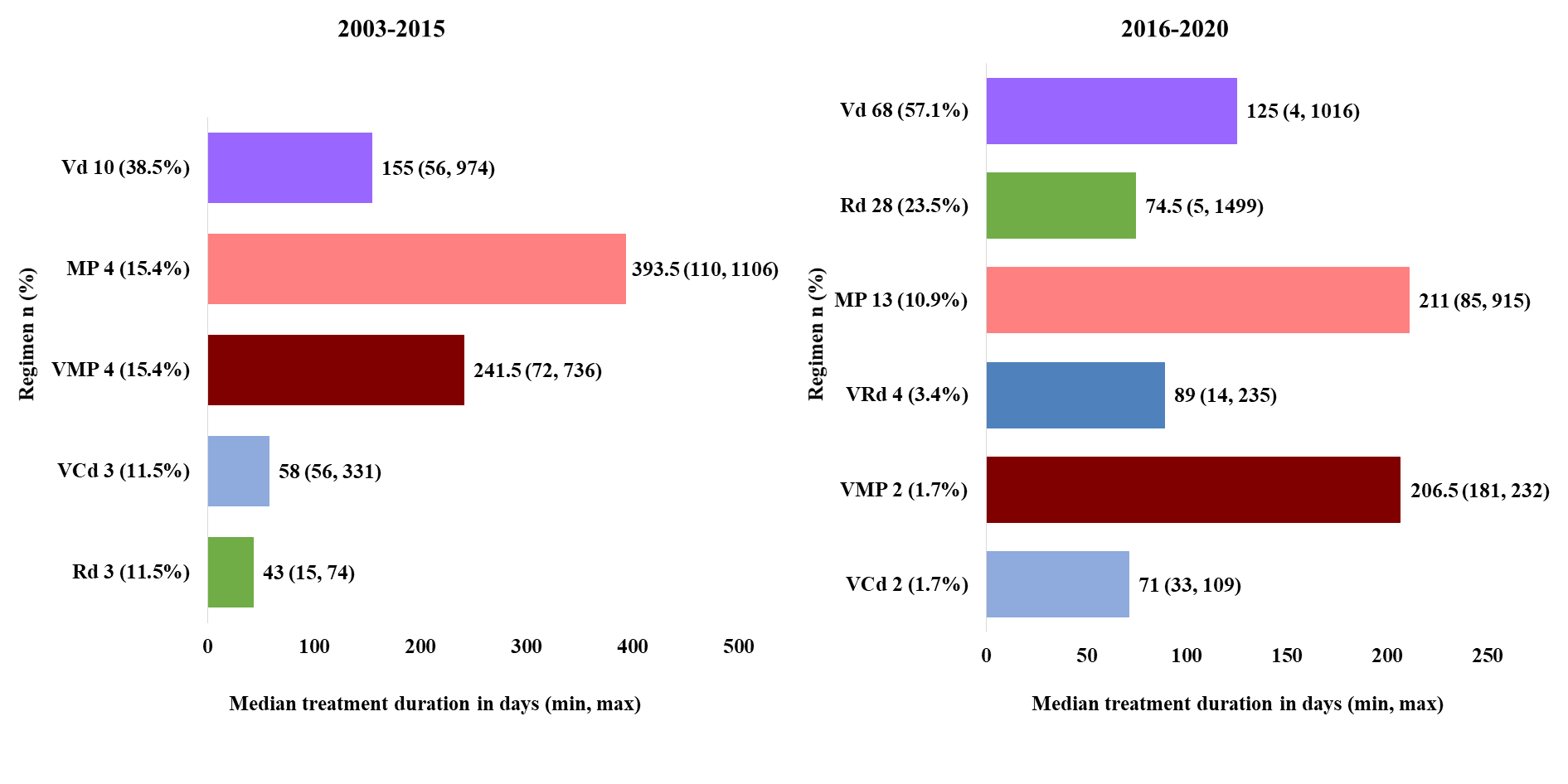


MP, melphalan/prednisolone; Rd, lenalidomide/dexamethasone; SCT, stem cell transplantation; VCd, bortezomib/cyclophosphamide/dexamethasone; Vd, bortezomib/dexamethasone; VMP, bortezomib/melphalan/prednisolone; VRd, bortezomib/lenalidomide/dexamethasone

**Figure S3B:** First-line treatment duration by selected regimen in SCT not-conducted group (without renal impairment)


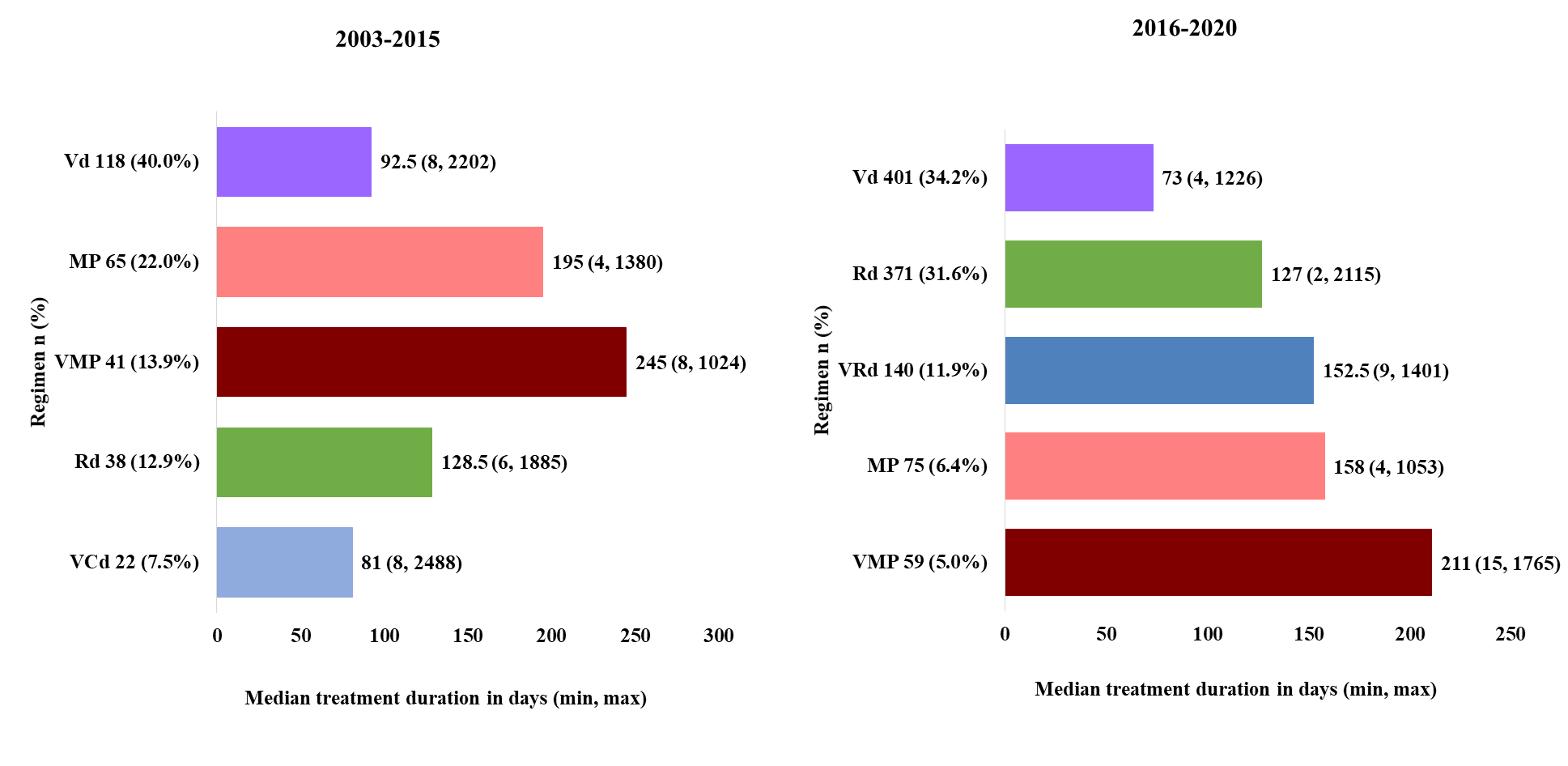


MP, melphalan/prednisolone; Rd, lenalidomide/dexamethasone; SCT, stem cell transplantation; VAd, bortezomib/adriamycin/dexamethasone; VCd, bortezomib/cyclophosphamide/dexamethasone; Vd, bortezomib/dexamethasone; VMP, bortezomib/melphalan/prednisolone; VRd, bortezomib/lenalidomide/dexamethasone

**Figure S4A:** Regimen selections for the lines of treatment in the SCT not-conducted group (2003–2015)


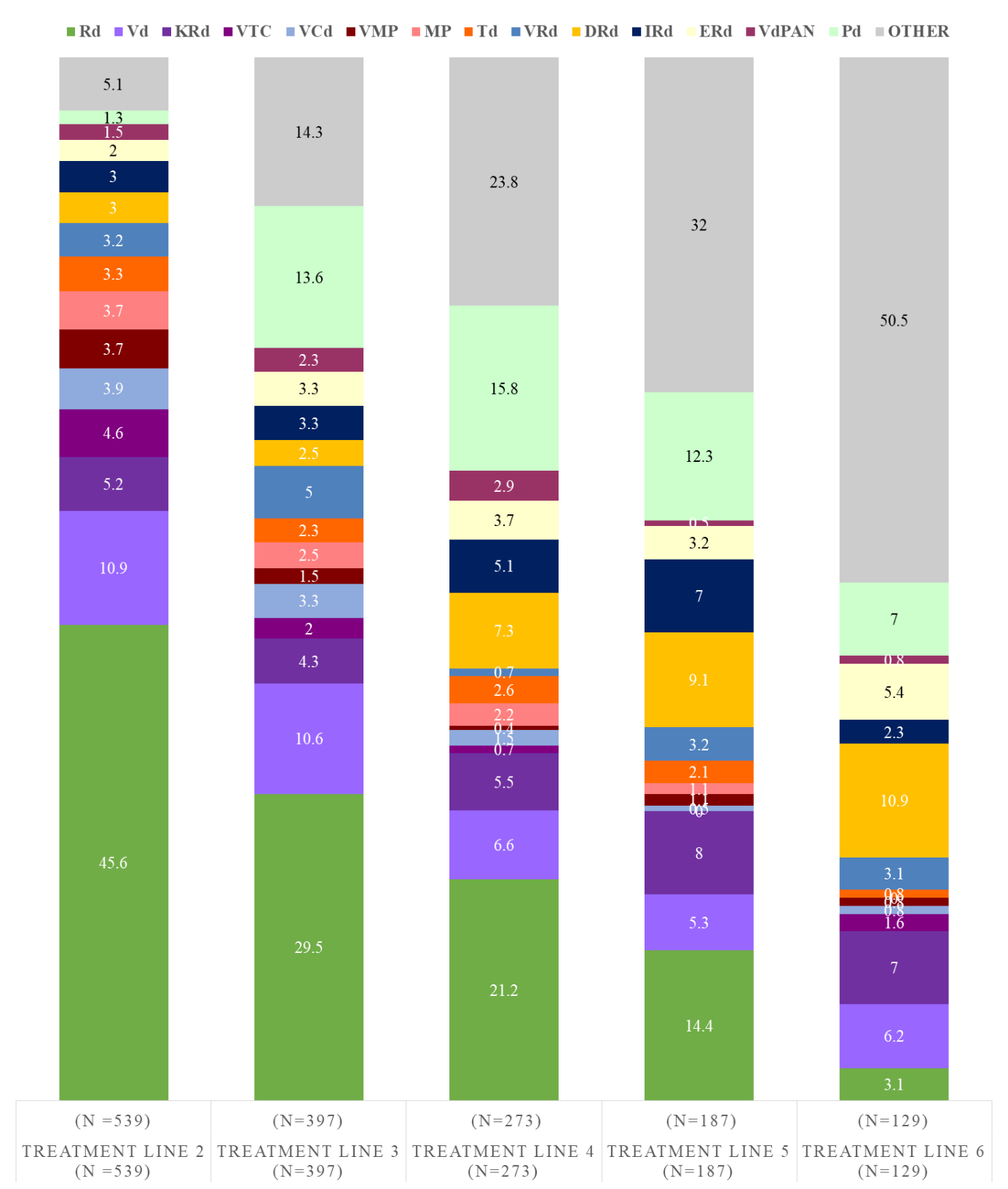


DRd, daratumumab/lenalidomide/dexamethasone; ERd, elotuzumab/lenalidomide/dexamethasone; IRd, ixazomib/lenalidomide/dexamethasone; KRd, carfilzomib/lenalidomide/dexamethasone; MP, melphalan/prednisolone; Pd, pomalidomide/dexamethasone; Rd, lenalidomide/dexamethasone; Td, thalidomide/dexamethasone; VCd, bortezomib/cyclophosphamide/dexamethasone; Vd, bortezomib/dexamethasone; VdPAN, bortezomib/dexamethasone/panobinostat; VMP, bortezomib/melphalan/predonisolone; VRd, bortezomib/lenalidomide/dexamethasone


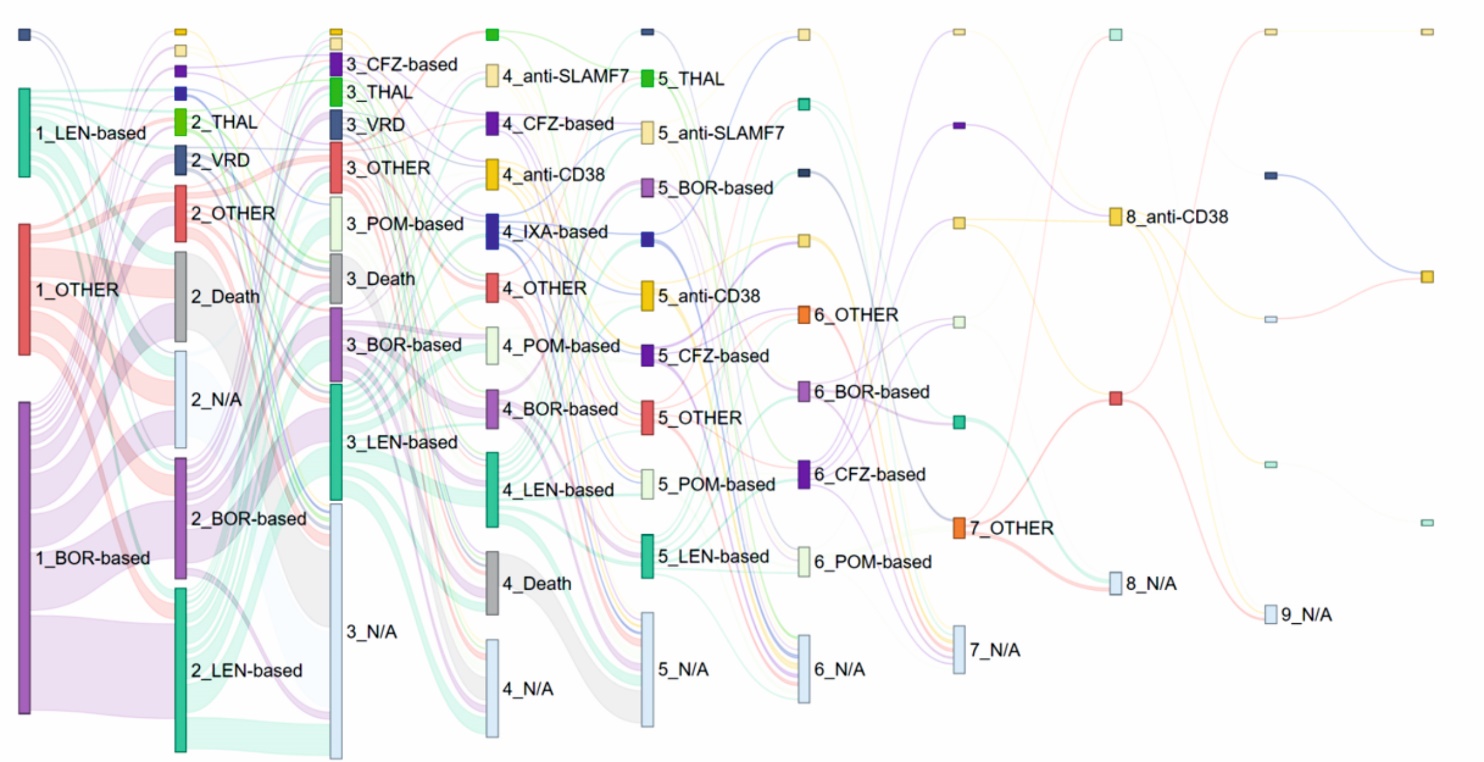


Anti-CD38, daratumumab; anti-SLAMF7, Elotuzumab; BOR-based, bortezomib-based regimen; LEN-based, lenalidomide based regimen; CFZ-based regimen, carfilzomib-based regimen; IXA-based, ixazomib-based regimen; N/A, No drug change; POM-based, pomalidmide-based regimen

**Figure S4B:** Regimen selections for the lines of treatment in the SCT not-conducted group (2016–2020)


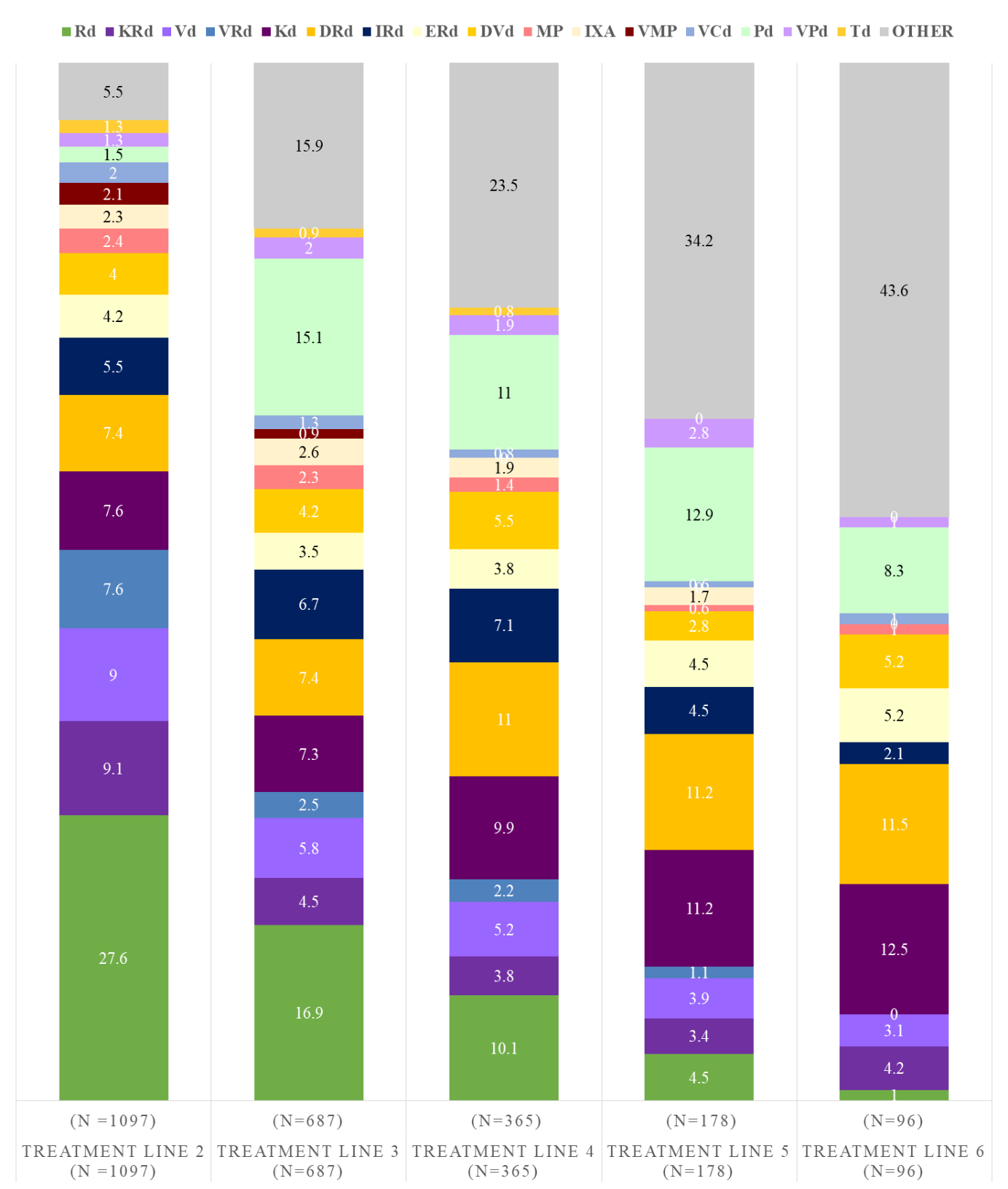


DRd, daratumumab/lenalidomide/dexamethasone; DVd, daratumumab/bortezomib/dexamethasone; ERd, elotuzumab/lenalidomide/dexamethasone; IRd, ixazomib/lenalidomide/dexamethasone; IXA, ixazomib; Kd, carfilzomib /dexamethasone; KRd, carfilzomib/lenalidomide/dexamethasone; MP, melphalan/prednisolone; Pd, pomalidomide/dexamethasone; Rd, lenalidomide/dexamethasone; Td, thalidomide/dexamethasone; VCd, bortezomib/cyclophosphamide/dexamethasone; Vd, bortezomib/dexamethasone; VMP, bortezomib/melphalan/ predonisolone; VRd, bortezomib/lenalidomide/dexamethasone


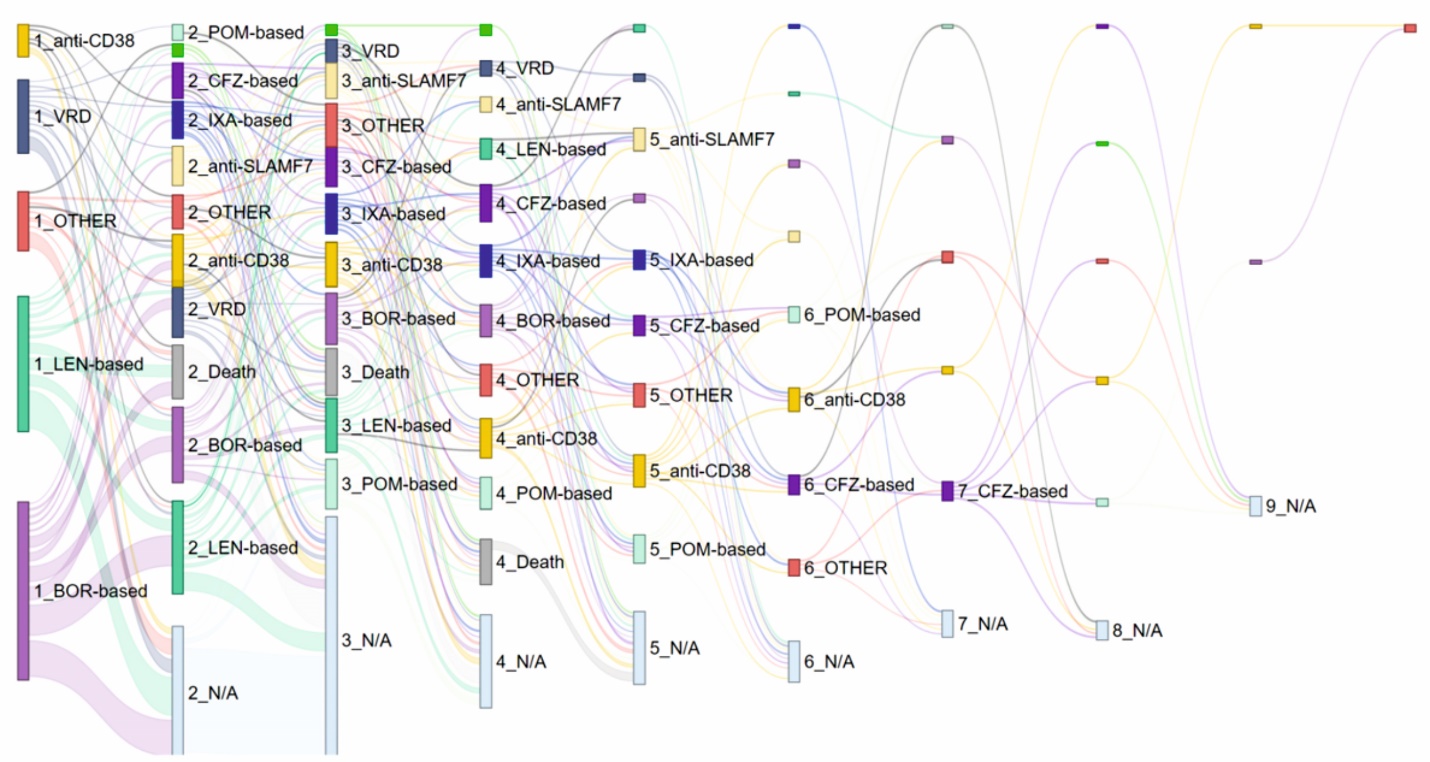


Anti-CD38, daratumumab; anti-SLAMF7, Elotuzumab; BOR-based, bortezomib-based regimen; CFZ-based regimen, carfilzomib-based regimen; IXA-based, ixazomib-based regimen; LEN-based, lenalidomide based regimen; N/A, No drug change; POM-based, pomalidomide-based regimen
